# Potency-Enhancing Mutations of Gating Modifier Toxins for the Voltage-Gated Sodium Channel Na_V_1.7 Can Be Predicted Using Accurate Free-Energy Calculations

**DOI:** 10.3390/toxins13030193

**Published:** 2021-03-07

**Authors:** Dana Katz, Dan Sindhikara, Michael DiMattia, Abba E. Leffler

**Affiliations:** Schrӧdinger, Inc., 120 West 45th St., New York, NY 10036, USA; dana.katz@schrodinger.com (D.K.); daniel.sindhikara@schrodinger.com (D.S.); michael.dimattia@schrodinger.com (M.D.)

**Keywords:** gating-modifier toxin, free-energy perturbation, sodium channel, drug discovery

## Abstract

Gating modifier toxins (GMTs) isolated from venomous organisms such as Protoxin-II (ProTx-II) and Huwentoxin-IV (HwTx-IV) that inhibit the voltage-gated sodium channel Na_V_1.7 by binding to its voltage-sensing domain II (VSDII) have been extensively investigated as non-opioid analgesics. However, reliably predicting how a mutation to a GMT will affect its potency for Na_V_1.7 has been challenging. Here, we hypothesize that structure-based computational methods can be used to predict such changes. We employ free-energy perturbation (FEP), a physics-based simulation method for predicting the relative binding free energy (RBFE) between molecules, and the cryo electron microscopy (cryo-EM) structures of ProTx-II and HwTx-IV bound to VSDII of Na_V_1.7 to re-predict the relative potencies of forty-seven point mutants of these GMTs for Na_V_1.7. First, FEP predicted these relative potencies with an overall root mean square error (RMSE) of 1.0 ± 0.1 kcal/mol and an R^2^ value of 0.66, equivalent to experimental uncertainty and an improvement over the widely used molecular-mechanics/generalized born-surface area (MM-GB/SA) RBFE method that had an RMSE of 3.9 ± 0.8 kcal/mol. Second, inclusion of an explicit membrane model was needed for the GMTs to maintain stable binding poses during the FEP simulations. Third, MM-GB/SA and FEP were used to identify fifteen non-standard tryptophan mutants at ProTx-II[W24] predicted in silico to have a at least a 1 kcal/mol gain in potency. These predicted potency gains are likely due to the displacement of high-energy waters as identified by the WaterMap algorithm for calculating the positions and thermodynamic properties of water molecules in protein binding sites. Our results expand the domain of applicability of FEP and set the stage for its prospective use in biologics drug discovery programs involving GMTs and Na_V_1.7.

## 1. Introduction

Voltage gated sodium channels (VGSCs) are transmembrane proteins with an essential role in electrical signaling, nerve conduction, skeletal and cardiac muscle contraction, secretion, and neurotransmission [1]. These ion channels are composed of a voltage-sensing alpha subunit that forms an ion-conducting pore and one or two beta subunits involved in channel expression and kinetics [2]. Nine distinct alpha subunits (Na_V_1.1 to Na_V_1.9) have been identified in humans and their dysregulation is associated with distinct disease phenotypes [3]. The alpha subunit is composed of four homologous domains (DI to DIV) each consisting of six transmembrane helical segments termed S1 to S6 [2] that organize into a pseudo-tetramer with each S1–S4 bundle forming a voltage sensing domain (VSD) and each set of S5-S6 helices contributing to the formation of a central pore. Extensive pharmacogenetic data suggest that inhibiting Na_V_1.7 activity should present a therapeutic approach for non-opioid analgesia [4], but challenges include developing an antagonist that is not only potent but also selective [5]. This is particularly important because Na_V_1.7 inhibitors with limited selectivity over other Na_V_ subtypes have an array of undesirable side effects [6]. Targeting the VSD, which is less conserved between VGSCs than the central pore [6], could be an effective strategy for achieving this goal [7].

**Figure 1:**
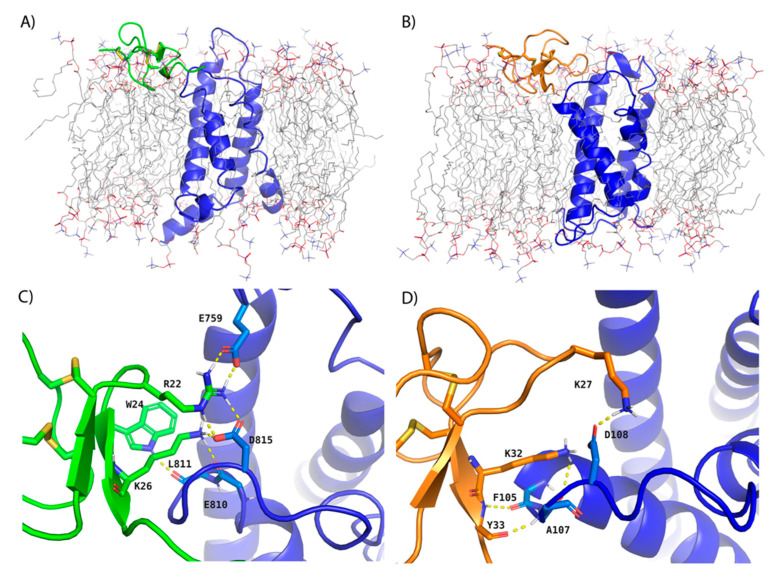
Cryo-EM-derived models of ProTx-II and HwTx-IV GMTs in complex with VSDII of Na_V_1.7 after MD simulation used for RBFE simulations. Depictions of the (**A**) transmembrane view of ProTx-II and Na_V_1.7 VSDII (**B**) transmembrane view of HwTx-IV and Na_V_1.7 VSDII (**C**) binding interface of ProTx-II and Na_V_1.7 VSDII and (**D**) binding interface of HwTx-IV and Na_V_1.7 VSDII after equilibration with 20 ns MD simulations in explicit membrane and solvent. ProTx-II, HwTx-IV, and Na_V_1.7 VSDII are depicted in cartoon representation and colored green, orange, and blue, respectively. The POPC membrane is shown as sticks with carbons colored gray. Hydrogen bonds between interacting residues at the protein:peptide interface are shown as dashed, yellow lines. Solvent molecules are omitted for clarity. The ProTx-II and Na_V_1.7 VSDII model is based on PDB entry 6N4R and the HwTx-IV and Na_V_1.7 VSDII model is based on PDB entry 6W6O.

Consequently, gating modifier toxins (GMTs) isolated from venomous animals have emerged as a leading modality for drugging Na_V_1.7 potently and selectively [8] as they inhibit VGSCs by binding to the VSDs to interfere with normal channel gating [9]. Protoxin-II (ProTx-II, a thirty-residue peptide) and Huwentoxin-IV (HwTx-IV, a thirty-five-residue peptide), derived from the tarantula *Thrixopelma pruriens* and the spider *Selenocosmia huwena*, respectively, are two such GMTs that have been pursued as Na_V_1.7 therapeutics [10,11]. Both are inhibitory cystine knot (ICK) peptides with three disulfide bonds that bind to VSDII of Na_V_1.7 to trap the channel in a non-conducting conformation [12,13]. Despite the promise of these GMTs, improving their potency by brute-force mutagenesis is difficult and costly, as evidenced by three studies in which few point mutations made to the toxins substantially improved potency for Na_V_1.7 [10,11,14]. Alternatively, recently determined cryo-electron microscopy (cryo-EM) structures of bacterial-human chimeric VGSCs in complex with ProTx-II and HwTx-IV and VSDII of Na_V_1.7 in the presence of phospholipids could be used in conjunction with structure-based methods to guide improvements to GMT potency [11,15]. These structures, in keeping with prior biophysical studies [16,17,18], establish that the GMTs form a “ternary complex” with the channel and membrane, utilizing an amphipathic surface to partition into the lipid bilayer that orients and stabilizes the toxin peptide for optimal interaction with VSDII of Na_V_1.7 via the extracellular membrane leaflet (Figure 1) [11,15,19]. Notwithstanding their significance, challenges remain for their use in structure-based drug design efforts. For example, the local resolution of the ProTx-II and HwTx-IV toxins was generally too poor to resolve side chains, owing in part to the difficulty of reproducing a native phospholipid bilayer environment in the context of membrane protein structure determination.

**Figure 2:**
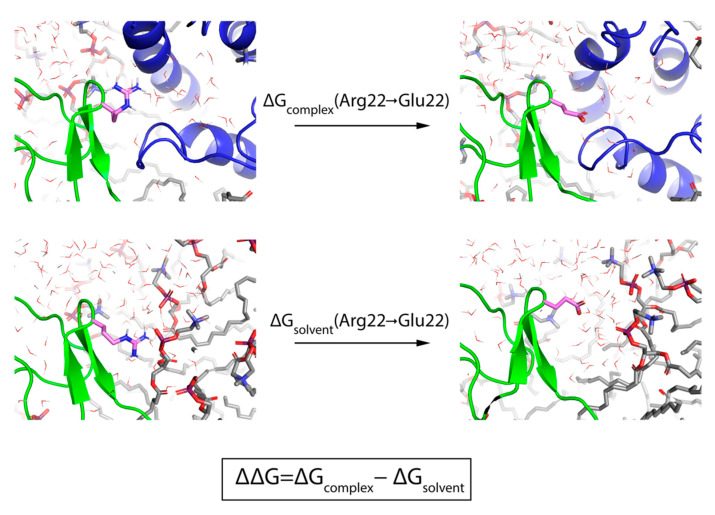
Prediction of relative potency of a GMT mutant with FEP. The thermodynamic cycle used for FEP calculations is shown using the mutation of ProTx-II[R22E] as an illustrative example. The top two panels show the mutation occurring in the “complex leg” of the simulation in which ProTx-II, VSDII, the membrane, and solvent molecules are present, while the bottom two panels show the mutation occurring in the “solvent leg” of the simulation in which only ProTx-II, the membrane, and solvent molecules are included. In all panels, ProTx-II is rendered as a green cartoon, Na_v_1.7 VSDII as a blue cartoon, and the mutated residue as sticks with pink carbons. In addition, POPC lipid molecules are shown as sticks with gray carbons and water molecules as lines with oxygen colored red and hydrogens colored white.

Computational techniques have the potential to utilize these cryo-EM structures to both guide where and what mutations should be made on a GMT. A recent study used WaterMap—a method which predicts the location, occupancy, and thermodynamics of water molecules—to partially explain the potency of ProTx-II in terms of its displacement of waters from unstable water sites near VSDII [20]. In addition, computational relative binding free-energy methods (RBFE) hold great promise for predicting how a mutation to a GMT will affect its potency for Na_V_1.7. Free energy perturbation (FEP) is a RBFE technique that uses all-atom molecular dynamics (MD) simulations to estimate the experimentally determined free energy change between an initial and final state (ΔΔG_EXP_), in this case wildtype (WT) GMT to mutant GMT, by simultaneously simulating these two states and a ladder of intermediates and summing the differential free energies between neighbors in complex and solvent (Figure 2) [21]. When there is sufficient overlap between adjacent intermediate states, FEP has been rigorously shown to calculate the overall relative predicted change in binding affinity (ΔΔG_FEP_) [22]. Throughout the simulation, FEP retains all degrees of freedom to account for conformational variation in ligand-receptor interactions and permits the displacement and introduction of explicit waters [23], which can result in a significant increase in accuracy over other predictive methods, such as Molecular mechanics-generalized born/surface area (MM-GB/SA), in which the protein is fixed and an implicit representation of the solvent is used [24,25]. The major drawback of FEP until recently was that it was computationally costly [26]. However, recent implementations of FEP that run on graphical processor units (GPUs) are significantly faster [27] and two recent studies found that FEP could predict the ΔΔG_EXP_ for dozens of mutations made to interacting proteins [25] and neutralizing antibodies [21] with a root-mean-squared error (RMSE) and mean-unsigned error (MUE) approaching the experimental limit of 1 kcal/mol. Despite their therapeutic relevance, no such large-scale FEP study has yet been carried out for GMTs and Na_V_1.7.

**Figure 3:**
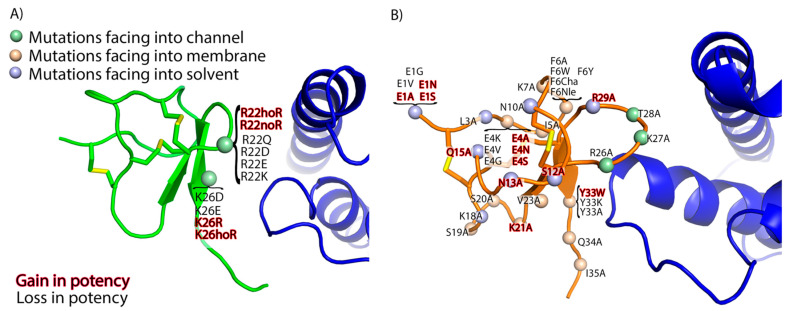
Simulated mutations. Mutations to (**A**) ProTx-II (green cartoon) and (**B**) HwTx-IV (orange cartoon) simulated in this study are shown. In both panels, Na_v_1.7 VSDII is shown as a blue cartoon. The Cɑ of each mutated residue is shown as a sphere colored light green if the sidechain of the residue is facing into VSDII, tan if facing into the membrane, and light blue if facing into solvent. Mutations that have a loss in potency are labeled in black and a gain in potency are labeled in red. Disulfide bonds are depicted with yellow sticks. The POPC membrane and solvent molecules are omitted for clarity.

Here, we explore the utility of FEP to predict changes in potencies of GMT mutants and as a means to optimize in silico the design of GMT-based therapeutics targeting voltage-gated sodium channels using the cryo-EM structures of ProTx-II and HwTx-IV in complex with Na_V_1.7. We hypothesize that it will be necessary to include an explicit membrane lipid bilayer (in effect, modeling the “ternary complex”) in order to accurately model GMT potency for Na_V_1.7. Further, we acknowledge several reasons why this system could be challenging for FEP, including the relatively poor local resolution of the toxin chains in the cryo-EM structures, the possible requirement of including an explicit membrane to model the ternary complex, and the large size and structural complexity of GMTs. Nevertheless, we begin by performing a retrospective study on forty-seven GMT mutants (Figure 3), re-predicting their experimentally measured changes in potency for Na_V_1.7 using both FEP and MM-GB/SA [10,11,14]. The dataset includes a large number of charge-change mutations, which historically have been challenging for FEP due to the fact that the peptide toxin binding mode can become unstable during these perturbations [25,28] (Figure 3). Next, to test our hypothesis regarding the importance of modeling the ternary complex for FEP calculations, we examine the pose stability of GMTs during FEP simulations with and without the membrane. Finally, we present a workflow consisting of WaterMap, MM-GB/SA, and FEP that can identify non-standard amino acid (NSAA) mutations predicted to cause an increase in potency. This is a proof-of-concept work that defines an extended domain of applicability for FEP and sets the stage for future prospective studies in which FEP can be used to rapidly and accurately identify potent and diverse mutations to GMTs targeting ion channels such as Na_V_1.7.

## 2. Results

### 2.1. Retrospective Performance of FEP on Mutagenesis Data

#### 2.1.1. Overall Performance

**Figure 4:**
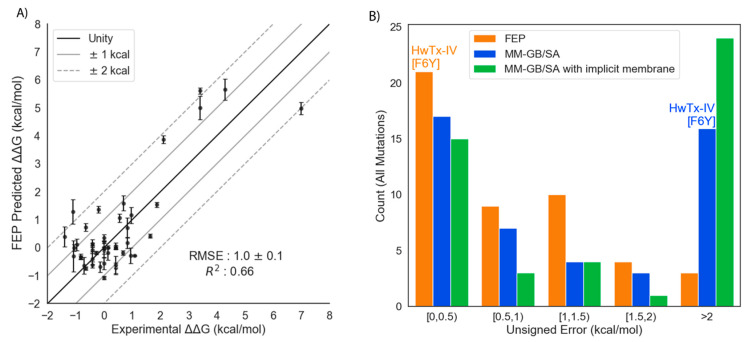

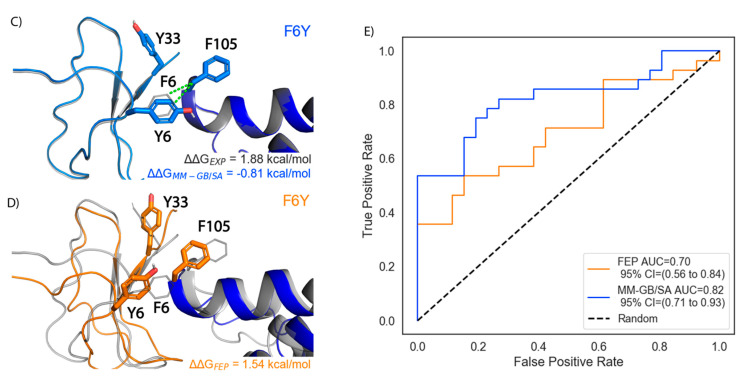
Overall retrospective FEP and MM-GB/SA performance. (**A**) Scatter plot of ΔΔG_FEP_ against ΔΔG_EXP_ with unity (solid, black line), ±1 kcal/mol error bands (solid, gray lines) and ±2 kcal/mol error bands (dashed, gray lines) superimposed. The error bars show the standard error of the mean (SEM) from three, independent FEP simulations (**B**) Histogram of unsigned errors for FEP (orange), MM-GB/SA (blue), and MM-GB/SA with an implicit membrane (green). The bins that the HwTx-IV[F6Y] prediction falls into for FEP and MM-GB/SA are labeled (**C**) Representative structure of HwTx-IV[F6Y] (light blue cartoon) from MM-GB/SA. VSDII is shown in dark blue cartoon rendering. Dashed green lines show hydrophobic contacts. Starting structure is in gray cartoon (**D**) Representative structure of HwTx-IV[F6Y] (orange cartoon) from FEP. Membrane and solvent molecules omitted for clarity (**E**) ROC plot illustrating the ability of FEP and MM-GB/SA to classify mutations having a relative gain in potency.

The performance of FEP was tested on 47 ProTx-II and HwTx-IV mutants that were previously synthesized and whose potency for human Na_V_1.7 had been measured using electrophysiology. FEP was able to re-predict the binding free energies of these mutants relative to the WT peptides with an overall RMSE of 1.0 ± 0.1 kcal/mol, and an R^2^ of 0.66 (Figure 4A and Table 1). Overall, 64% of the FEP predictions were within 1 kcal/mol of the experimental measurements, 30% were between 1 and 2 kcal/mol, and 6% were off by more than 2 kcal/mol. In contrast, for MM-GB/SA the RMSE was 3.89 ± 0.82 kcal/mol and the R^2^ was 0.56 (Table 1). Rescaling the MM-GB/SA by a constant resulted in an improved RMSE of 1.50 ± 0.28 kcal/mol but poorer R^2^ of 0.44. To remain consistent with previous MM-GB/SA studies of peptide toxins and ion channels, only unscaled results are included in this work [29]. Figure 4B shows the distribution of unsigned errors for FEP, MM-GB/SA, and MM-GB/SA with an implicit membrane. Out of 47 mutations, FEP predicted 30 with an unsigned error of less than 1 kcal/mol and 7 mutations with an unsigned error greater than 1.5 kcal/mol. Conversely, MM-GB/SA with and without an implicit membrane predicted 19 and 25 mutations, respectively, with an error greater than 1.5 kcal/mol.

One activity cliff mutation in this dataset, HwTx-IV[F6Y], has a significant +1.88 kcal/mol loss in affinity for Na_V_1.7 despite featuring only a modest chemical modification, the addition of a hydroxyl group. FEP correctly classified this mutation as causing a loss in potency with a predicted ΔΔG of 1.54 kcal/mol that was within 0.34 kcal/mol of the experimental value. In contrast, MM-GB/SA predicted a ΔΔG of -0.81 kcal/mol and MM-GB/SA with an implicit membrane predicted a ΔΔG of −0.43 kcal/mol. Both of those predictions misclassified a loss in potency mutation as having a gain in potency. To understand these discrepant predictions, we inspected the MM-GB/SA and FEP representative poses (Figure 4C,D). The MM-GB/SA pose had a slight movement of Tyr-6, which retained its hydrophobic interaction with Phe-105 as was present in the native (Figure 4C). In contrast, the GMT:Na_V_1.7 interface underwent more substantial conformational changes in FEP, with HwTx-IV[F6Y] losing its hydrophobic interactions with both Tyr-33 and Phe-105 (Figure 4D). The loss of this interaction as well as increased movement in side chains could largely account for the correctly predicted loss in potency by FEP.

We also explored the ability of FEP to act as a binary classifier that could distinguish between mutations that cause a gain in potency (ΔΔG_EXP_ < 0) versus those that cause a loss in potency (ΔΔG_EXP_ > 0) (Figure 4E). The FEP Receiver Operating Characteristic (ROC) curve had an Area Under the Curve (AUC) of 0.70 and a 95% confidence interval (CI) of 0.56 to 0.84, which was statistically significant with a *p*-value of 0.02. MM-GB/SA had an AUC of 0.82 and a 95% CI of 0.71 to 0.93. FEP classified mutations with a gain in potency with 61% accuracy compared to MM-GB/SA that classified mutations with a 76% accuracy. MM-GB/SA with an implicit membrane had an accuracy of 55%, although its AUC was 0.80 with a 95% CI of 0.67 to 0.92. These results indicate that both FEP and MM-GB/SA performed better than random (AUC = 0.5) when classifying mutations that have a gain in potency.

#### 2.1.2. Performance by GMT and Physicochemical Category

For ProTx-II and HwTx-IV, FEP was able to recapitulate the binding free energies with an RMSE of 1.7 ± 0.16 kcal/mol and 0.69 ± 0.07 kcal/mol, respectively, with corresponding R^2^ values of 0.78 and 0.19, respectively (Table 1). MM-GB/SA predicted the binding free energies for ProTx-II and HwTx-IV with an RMSE of 7.57 ± 1.48 kcal/mol and 1.95 ± 0.64 kcal/mol, with R^2^ values of 0.68 and 0.24, respectively. The performance of both methods was also broken down by physicochemical property. For charge change mutations, FEP outperformed MM-GB/SA with an RMSE of of 0.96 ± 0.15 kca/mol and an R^2^ of 0.81, compared to MM-GB/SA which had an RMSE of 3.75 ± 1.3 kcal/mol and a comparable R^2^ of 0.79 (Table 1). For neutral mutations in which the charge was conserved, FEP had an RMSE of 1.02 ± 0.14 kcal/mol whereas MM-GB/SA had an RMSE of 4.04 ± 1.3 kcal/mol (Table 1).

#### 2.1.3. Performance for Mutations with Proximity to the Membrane

**Figure 5:**
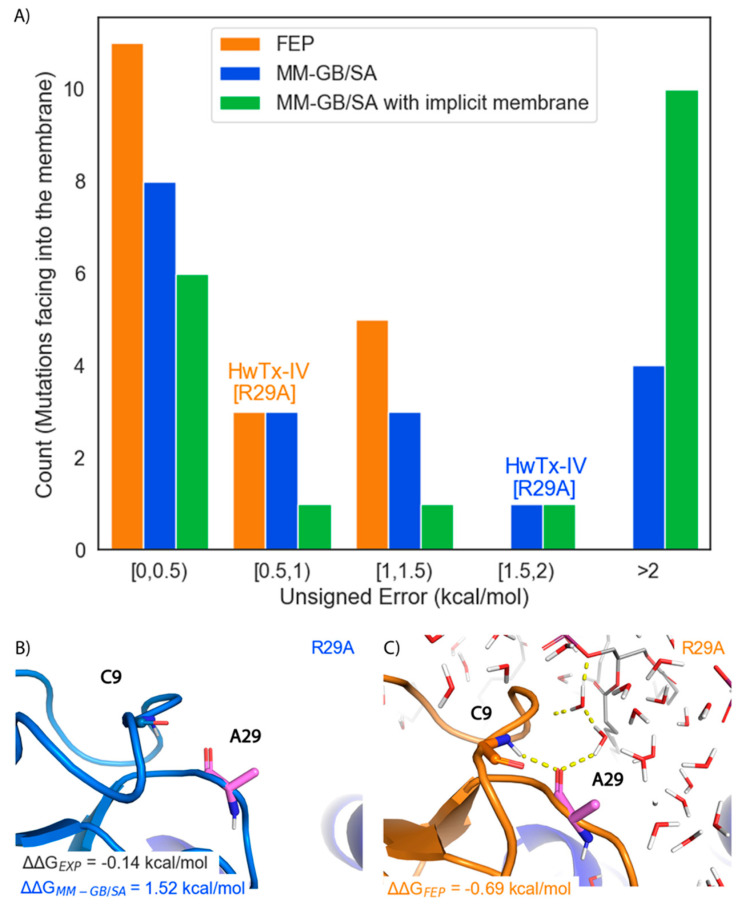
Retrospective performance of FEP and MM-GB/SA on membrane facing mutations. (**A**) Histogram of Unsigned Errors for FEP (orange), MM-GB/SA (blue), and MM-GB/SA with an implicit membrane (green). The bins that the HwTx-IV[R29A] prediction falls into for FEP and MM-GB/SA are labeled (**B**) Representative structure for HwTx-IV[R29A] (light blue cartoon) from the MM-GB/SA simulation (**C**) Representative structure for HwTx-IV[R29A] (orange cartoon) from the FEP simulation. Carbon atoms of the mutated residue are colored pink and VSDII is shown as a dark blue cartoon. Hydrogen bonds are indicated with dashed yellow lines. POPC membrane is shown in sticks with carbons colored gray. Water molecules are shown as lines with oxygens colored red and hydrogens colored white.

Due to the hypothesized importance of the membrane in the “ternary complex,” we examined its influence on the free-energy calculations in depth. There were nineteen mutations to residues that face into the membrane. Overall, FEP predicted potency changes for this subset with high accuracy as indicated by an RMSE of 0.71 ± 0.10 kcal/mol and an R^2^ of 0.31 (Table 1). For MM-GB/SA, the RMSE was 1.65 ± 0.32 kcal/mol with an R^2^ of 0.25 (Table 1). Surprisingly, the addition of an implicit membrane to the MM-GB/SA calculations worsened the predictions, with the RMSE increasing to 3.16 ± 1.0 kcal/mol and an R^2^ of 0.006. FEP predicted 11 out of the 19 mutations with an unsigned error less than 0.5 kcal/mol and no mutations with an error above 1.5 kcal/mol (Figure 5A). MM-GB/SA predicted eight mutations with an error less than 0.5 kcal/mol and five mutations with an error larger than 1.5 kcal/mol (Figure 5A). MM-GB/SA with an implicit membrane predicted six mutations with an error less than 0.5 kcal/mol and eleven mutations with an error larger than 1.5 kcal/mol (Figure 5A).

The HwTx-IV[R29A] mutation that has ΔΔG_EXP_ of −0.14 kcal/mol was predicted well by FEP with a ΔΔG of −0.69 kcal/mol, but poorly by MM-GB/SA with a ΔΔG of 1.52 kcal/mol. To rationalize the difference in these predictions, the poses of this mutant produced by both methods were examined (Figure 5B,C). Due to the absence of explicit solvent molecules in MM-GB/SA, HwTx-IV[A29] rotated inwards to try and make a hydrogen bond with the amine backbone of Cys-9, leading to a highly unfavorable unsatisfied hydrogen bond (Figure 5B). In contrast, in FEP, HwTx-IV[A29] was oriented such that a hydrogen bond was formed between the backbone carbonyl of Ala-29 and amine of Cys-9, leading to a hydrogen bond network between Ala-29, Cys-9 and solvent molecules (Figure 5C). In turn, these solvent molecules formed additional hydrogen bonds with the hydrophilic headgroup of the lipid bilayer.

### 2.2. Stability of GMT:VSD Complex during FEP Simulations

**Figure 6:**
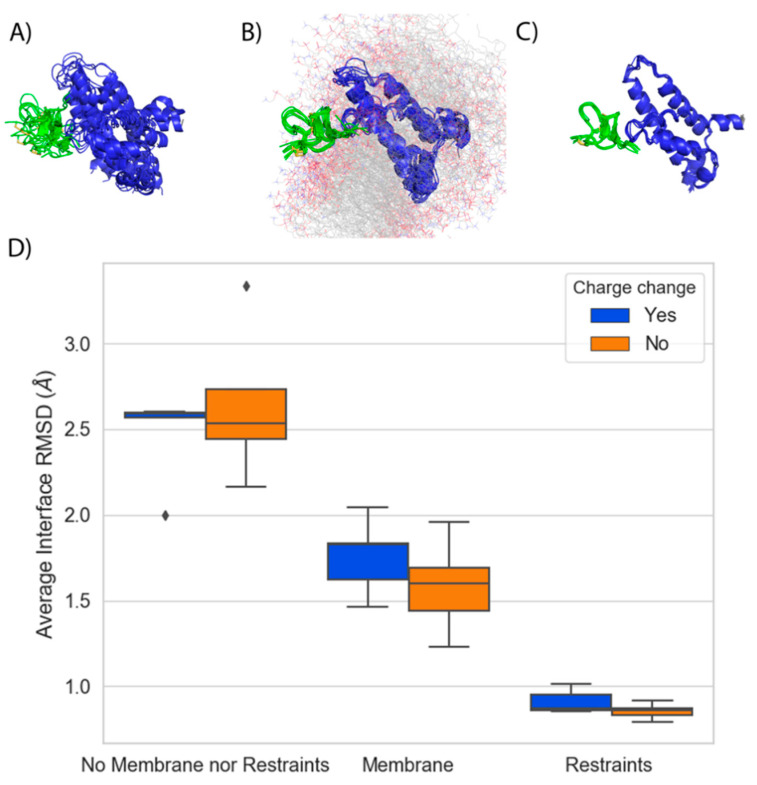
ProTx-II pose stability during FEP simulations. Aligned snapshots of WT ProTx-II (green cartoon) and VSDII (blue cartoon) extracted from FEP simulations with (**A**) neither a membrane nor restraints (**B**) a membrane only (**C**) Cɑ position restraints only. Water molecules are omitted for clarity. The membrane is shown in line representation with carbons colored gray, oxygen colored red, phosphorus purple, and nitrogen blue. (**D**) Boxplots summarizing the mean iRMSD over the last 5ns of FEP simulations performed using the conditions described in (**A**)–(**C**) for the ten ProTx-II mutants. Mutations in which the charge is changed are grouped in the blue boxplot, whereas mutations for which the charge is conserved are grouped in the orange boxplot.

A stable binding mode for the GMT is necessary for accurate FEP predictions as shown in previous studies [25,28]. We examined the pose stability of ten ProTx-II mutants for three different kinds of FEP simulations: no membrane present, membrane present, and Cɑ position restraints present. When neither membrane nor restraints were present, aligned snapshots from the trajectory revealed that during these MD simulations the GMT and VSDII experienced a large amount of flexibility, causing the protein-protein interface to change throughout the simulation. Importantly, the GMT itself also rotated and translated between frames, suggesting a stable binding mode was not achieved for this system (Figure 6A). The addition of the membrane stabilized the backbone of the peptide to the degree that the pose of the GMT remained stable, but still allowed for movement of side chains to find alternate conformations (Figure 6B). Finally, the addition of position restraints, as intended, severely limited the motion of both the GMT and VSDII such that the simulation frames overlayed nearly identically (Figure 6C).

We quantified these observations by calculating the Root-Mean-Square-Deviation (RMSD) of the positions of the Cɑ atoms of the residues at the GMT and channel interface (iRMSD) over the final 5 ns of the FEP simulations. We found that when neither the membrane nor restraints were present, the average iRMSD was 2.56 Å (Figure 6D). The addition of the membrane stabilized the complexes on average, resulting in an iRMSD of 1.67 Å. Finally, using position restraints resulted in an average iRMSD of 0.88 Å. The addition of the membrane stabilized the complex enough such that even charge-change mutations preserved a stable binding mode with an average iRMSD of 1.8 Å, compared to 2.5 Å with no membrane present (Figure 6D). Taken together, these data suggest that the membrane acts to restrain the motion of the VSDII and the GMT and enable the GMT to maintain a stable binding pose during FEP simulations.

### 2.3. Improvement of ProTx-II Potency for Na_V_1.7 in Silico

**Figure 7:**
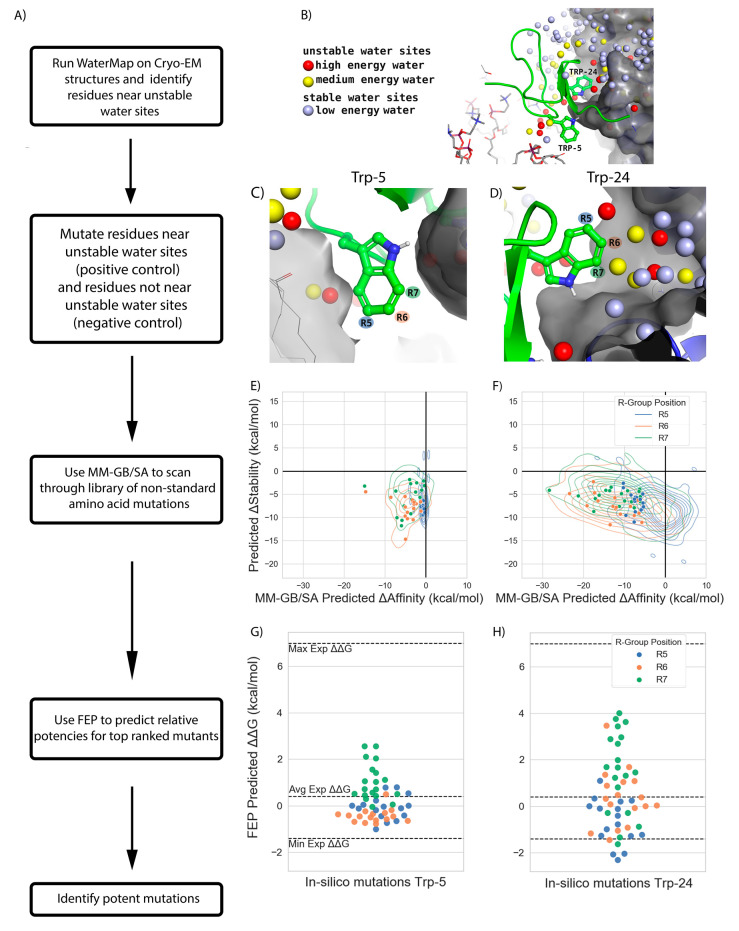
Workflow for in silico optimization of ProTx-II. (**A**) Description of each stage in the workflow. (**B**) Holo WaterMap of the ProTx-II and VSDII interface with water sites colored as described in the legend. (**C**) Water sites near negative control ProTx-II[W5] (**D**) Water sites near positive control ProTx-II[W24] (**E**) ProTx-II[W5] and (**F**) ProTx-II[W24] contour plots summarizing the MM-GB/SA predicted ΔStability vs. ΔAffinity when mutating either residue to each of 617 non-standard tryptophan analogues. Points indicate fifty-two mutations chosen to be simulated with FEP. Contour and points are colored by the position on the indole ring at which a substituent is present (**G**) ProTx-II[W5] and (**H**) ProTx-II[W24] jitter plots of FEP predicted relative potencies of fifty-two Trp analogues to which each residue was mutated.

Identifying mutations that can increase the affinity of a GMT for its ion channel target is the ultimate goal of most mutagenesis studies and RBFE methods should seek to enable this aim quickly and accurately. MM-GB/SA, a high throughput method, performed well at classifying mutations as possessing a gain or loss in affinity but dramatically overestimated the magnitude of these changes (Figure 4 and Table 1). FEP, while computationally demanding, quantitatively predicted the relative change in affinity of mutations with high accuracy (Figure 4 and Table 1). These results suggest that the two techniques could be combined into a workflow to identify GMT mutants predicted to have a gain in potency for the VGSC (Figure 7A).

For the first step of the workflow, we ran a WaterMap of ProTx-II bound to Na_V_1.7 to identify residues suitable for mutagenesis as has been performed previously [20]. WaterMap placed a total of 101 water sites within 5 Å of the protein-protein interface atoms (Figure 7B). Seventy sites were predicted to be stable (low-energy) and 31 were predicted to be unstable (medium-energy or high-energy). Of the unstable sites, twenty were medium-energy and eleven were high-energy (Figure 7B). Visual inspection of the WaterMap revealed that Trp-5 did not have any unstable water sites near its indole sidechain (Figure 7C), while Trp-24 was close to at least three unstable water sites located in a small, hydrophobic groove on VSDII (Figure 7D). These data suggest that for our workflow Trp-24 could serve as a “positive control,” as we do expect to find analogues of Trp at position 24 predicted to gain potency by displacing unstable waters, while Trp-5 could serve as a “negative control” as there are no unstable waters in its vicinity available to displace to gain potency.

For the second step of the workflow, MM-GB/SA was used to predict the change in binding affinity (ΔAffinity) and stability (ΔStability) when mutating ProTx-II[W24] or ProTx-II[W5] to each of 617 NSAA analogues of Trp modified at the R5, R6, or R7 position. Mutations were examined that had ΔStability < 0 to preclude unfolding and ΔAffinity < −5 kcal/mol to account for the tendency of MM/GB-SA to exaggerate affinities. At ProTx-II[W5] out of 617 mutations, 149 mutations (24%) met these criteria with the lowest predicted ΔAffinity being −15 kcal/mol (Figure 7E). Conversely, ProTx-II[W24] had 371 such mutations (60%), with the lowest predicted ΔAffinity being −32 kcal/mol (Figure 7F). The predicted ΔAffinity and ΔStability distributions also differed between the R5, R6, and R7 positions. These data imply that modifications to Trp-24 would more likely lead to gains in potency than modifications to Trp-5.

Finally, the third step of the workflow consisted of testing a subset of mutations with FEP to get a more accurate prediction of their ΔΔGs. This subset included fifty-two mutations denoted by points in Figure 7E,F. ProTx-II[W5] only had one mutation with ΔΔG_FEP_ less than −1 kcal/mol, and none were predicted to be more potent than those that have already been experimentally characterized (Figure 7G). In contrast, at ProTx-II[W24] 21% of mutations had ΔΔG_FEP_ less than −1 kcal/mol, and three were predicted to have ΔΔG_FEP_ less than −2 kcal/mol, which would be a greater gain in potency than any experimentally measured mutation (Figure 7H). While experimental corroboration of these predictions is needed to draw definitive conclusions, in sum these results suggest that the workflow introduced here has the potential to identify GMT mutations that could improve potency for a VGSC.

## 3. Discussion

### 3.1. FEP Accurately Predicts the Relative Change in Potency of GMT Mutants for Na_V_1.7 Using Cryo-EM Structures

The principal finding from this study is that FEP can predict the change in potency of GMT mutants targeting VSDII of Na_V_1.7 with an RMSE of 1.0 kcal/mol and an R^2^ value of 0.7 (Figure 4 and Table 1). This RMSE and R^2^ were consistent with a well-validated FEP model [22]. They were also near the best performance that could be expected given the sample size, dynamic range, and experimental uncertainty in the dataset [30] and outperformed the widely used MM-GB/SA method (RMSE = 3.9 kcal/mol, R^2^ of 0.6). The performance of FEP is impressive given the significant sources of uncertainty in the study, such as the large size of GMTs, the relatively low resolution of the GMTs in the cryo-EM structures, and the fact that functional (electrophysiological) as opposed to binding data were also employed for benchmarking. FEP performed well for charge-change mutations with an RMSE of 0.96 ± 0.15 kcal/mol and an R^2^ of 0.81. This category of mutations has historically been challenging for FEP [28], suggesting that recent improvements for predicting charge-changes [25] were effective. Taken together, these data suggest that FEP can be used to accurately predict how a mutation to a GMT that targets VSDII of Nav1.7 will affect its potency for the channel, setting the stage for its prospective application in biologics drug discovery programs.

Despite the excellent overall performance, for ProTx-II FEP predicted losses in potency for some mutations that experimentally have been shown to gain potency. Of these false negatives, ProTx-II[R22norR] and ProTx-II[K26R] were most notable. ProTx-II[R22norR] has a reported ΔΔG_EXP_ of −1.4 kcal/mol while ΔΔG_FEP_ was +0.40 kcal/mol, a misprediction of 1.8 kcal/mol with the incorrect sign. Similarly, ProTx-II[K26R] has a ΔΔG_EXP_ of −1.1 kcal/mol while ΔΔG_FEP_ was +1.29 kcal/mol, a misprediction of 2.39 kcal/mol with the incorrect sign. Interestingly, both of these experimental measurements are difficult to rationalize based on the cryo-EM structure and on other findings in the literature. Arg-22 is believed to antagonize S4 gating-charge movement through interactions with acidic residues such as Glu-810, Asp-815, and Glu-817 in the extracellular vestibule of VSDII [11]. As a result, it is difficult to understand how norR, whose alkyl chain is one carbon shorter than arginine and thus further from this acidic pocket, could actually be more potent given the rapid distance dependent drop-off in electrostatics. Similarly, an experimental study showed that an analogue of ProTx-II in which all the Lys residues were mutated to Arg had a ~30-fold loss in potency [19], which is to some extent at odds with the finding that ProTx-II[K26R] had a ~10-fold gain in potency. It is possible that the ProTx-II[R22norR] and ProTx-II[K26R] analogues may warrant re-measurement. Alternatively, these mutant peptides could be folding into a conformation that differs significantly from the WT GMT or inducing a conformational rearrangement in VSDII upon binding that is outside the scope of the protein motion sampled during the FEP simulations. These possibilities could be tested by obtaining cryo-EM structures with these peptides complexed to VSDII of Na_V_1.7.

Finally, the R^2^ value for HwTx-IV was lower than for ProTx-II. This may be attributable to not only a smaller dynamic range in potencies amongst the HwTx-IV mutants, but also to a larger uncertainty in the HwTx-IV-bound structure in which the VSDs adopt a surprising “up” conformation, even with HwTx-IV bound [15]. In keeping with the latter hypothesis, a very recent paper purports to have identified an alternate resting state structure of Na_V_1.7 bound to HwTx-IV, although it has not yet been made available [31]. Future work could focus on running FEP using this structure to see if the correlation between measured and predicted potencies is improved.

### 3.2. Including an Explicit Membrane in FEP Simulations Improves the Model

We found that inclusion of an explicit membrane in the FEP simulations had two benefits. First, the POPC membrane stabilizes ProTx-II analogously to a Cɑ position restraint (Figure 6B–D). This is an important discovery because one of the prerequisites for free-energy calculations is a consistent binding mode over the course of the simulation. Indeed, previous studies had identified the lack of a stable pose during FEP calculations, particularly when the mutation involved a change in charge [28], as a roadblock to the use of the method. The second benefit of including a membrane during protein FEP was that it allowed modelling of atomic interactions between the membrane, GMT, and waters to be captured during the simulation. For example, for HwTx-IV[R29A] membrane atoms interacted via water networks with the backbone carbonyl of residue 29 on HwTx, an important stabilizing effect (Figure 5C). Such energetic contributions, in the form of both the Coulomb and Lennard-Jones energy terms, were thus considered during the FEP calculation and may have contributed to the excellent RMSE of 0.7 kcal/mol for mutations that face into the membrane (Table 1). Surprisingly, our results suggest that addition of an implicit membrane to the MM-GB/SA calculations did not improve those predictions. It appears that even though at a coarse level an implicit membrane mimics the biological environment of the membrane by introducing a lower dielectric, this representation does not allow key, detailed atomic interactions to be considered. Given the important role that the membrane played in this study, subsequent work might focus on developing and using more realistic membrane models, especially since it has been shown that there is a correlation between membrane binding affinity of a GMT and its potency as a Na_V_1.7 inhibitor [16,19].

### 3.3. A Workflow Consisting of WaterMap, MM-GB/SA, and FEP Can Rapidly Identify NSAAs Predicted to Increase Potency of ProTx-II for Na_V_1.7 in Silico

The third key finding from this study is that a workflow consisting of WaterMap, MM-GB/SA, and FEP (Figure 7A) was able to identify in silico potency enhancing GMT mutations for ProTx-II. We performed an in silico “dry-run” with this workflow by mutating ProTx-II Trp-5 and Trp-24 to 617 different Tryptophan analogues. We chose these two positions as a “negative control” and “positive control”, respectively, because it would seem more likely to find potency boosting mutations for Trp-24, which faces into a groove on Na_V_1.7 ideal for gaining potency through displacement of waters from unstable water sites (Figure 7D), than for Trp-5 that sits at the interface of solvent and membrane where no such water sites are present (Figure 7C). Indeed, our results conform to these expectations: no mutations to Trp-5 were predicted to be more potent than those already known (Figure 7G), whereas five such predicted mutations were identified for Trp-24 (Figure 7H). In contrast to the months or even years that might be expended looking for potency boosting mutations in a trial-and-error fashion, these calculations required only a few days of compute time.

Rapidly scoring hundreds of mutations with confidence depended on the ability of MM-GB/SA to accurately classify mutations as having either a gain or loss in potency with an AUC of 0.82 and a 95% CI of 0.71 to 0.93 (Figure 4E), which is at the high end of previously reported MM/GB-SA performances [24,32]. It appears that the disulfide stabilized structures of GMTs [19] make them amenable to approximate RBFE scoring. That is, if the GMT is essentially acting as a “rigid-body” to dock into the VSDII deactivated state, then the good performance of MM-GB/SA is consistent with the fact MM-GB/SA does not account for significant conformational flexibility. Two recent studies in which molecular dynamics was used to simulate the HwTx-IV and Na_V_1.7 complex also found key interactions between the peptide and channel to be highly stable, lending credence to the notion that HwTx-IV might bind as rigid-body as well [33,34]. Ultimately, as additional cryo-EM structures of GMTs with ion channels emerge it will become clearer if ProTx-II and HwTx-IV are the exception or rule.

Although experimental validation would be needed to corroborate these predictions, given the excellent retrospective performance of both RBFE methods we suggest that a combination of WaterMap, MM-GB/SA, and FEP can be used as an efficient workflow to identify key residues, explore large numbers of standard and non-standard mutations, and accurately predict relative potencies for top ranked mutations to accelerate the development of peptides into ion channel therapeutics.

## 4. Materials and Methods

### 4.1. Protein Structure Preparation

All calculations were performed using the 2020-2 release of Maestro (Schrӧdinger, Inc.: New York, NY, USA) unless otherwise noted. Na_V_1.7 VSDII (deactivated state) in complex with ProTx-II and Na_V_1.7 VSDII chimera in complex with HwTx-IV were downloaded from the OPM database using their PDB codes, 6N4R and 6W6O, respectively. For 6N4R, the protein system was truncated to only include VSDII of Chain B and ProTx-II (Chain F), and for 6W6O the protein was truncated in a similar manner to only include VSDII from Chain F and Chain G for HwTx-IV. We truncated the system in this way because the chimeric structures used in this study are almost entirely bacterial in origin except for a subset of the human Na_V_1.7 VSDII sequence (which engages the GMTs) that was grafted onto the bacterial VSD backbone. By including only VSDII in our simulations, we are treating the VSD as a distinct structural unit, in line with previous structural studies of VSGCs, while also acknowledging that including more of the channel, such as the central pore domain that buttresses VSDII, may be inappropriate given its bacterial nature. For both the protein and peptides the Protein Preparation Wizard was used to cap the N- and C-termini with acetyl and N-methyl groups, respectively. Protonation states were assigned using PROPKA at pH 7.4. Hydrogen bond networks were optimized using the “H-bond assignment” panel. Restrained minimization was carried out using the OPLS3e force field [35] and heavy atoms converged to an RMSD of 0.3 Å. Next, using the System Builder panel, a predefined SPC solvent model and a POPC membrane model were placed on the pre-aligned structure. No neutralizing counterions or salt was added. Once the membrane was built onto the system, a molecular dynamics (MD) simulation with Desmond (Desmond Molecular Dynamics System, D. E. Shaw Research, New York, NY, USA, 2020. Maestro-Desmond Interoperability Tools, Schrödinger, New York, NY, USA, 2020) was performed for 20 ns on a GPU cluster consisting of NVIDIA GeForce GTX 1080 and 1080ti GPUs. The final frame from this simulation was extracted and used as input for FEP calculations.

### 4.2. Selection and Categorization of Mutants

Ten ProTx-II and thirty-seven HwTx-IV mutations were gathered from three sources [10,11,14] and all mutations with reported pK_i_ values were used for FEP benchmarking except for HwTx-IV[G36A] and HwTx-IV[K37A] because those residues are not included in the native peptide sequence. HwTx-IV[E1Pyr] (pyroglutamate) and HwTx-IV[F6(2Nal)] (2-Naphthylalanine) mutations were also excluded as they were not suitable for FEP calculations due to cyclization and steric clashes. Seven mutations with percentage inhibition values (but not pK_i_s) were additionally used for assessing classifier performance but not RMSEs as they could not readily be converted to ΔΔG_EXP_ values. Mutations were categorized by their location on the peptide and their physicochemical property. Residues that faced into the channel were identified using the Protein-Protein interface selection tool and residues that faced into the membrane were identified by the Protein-Membrane interface selection tool in Maestro. To convert reported IC_50_ values to ΔΔG_EXP_, the relation ΔΔG_EXP_ = R*T*ln(IC50_MUT_/IC50_WT_) was used in which IC50_MUT_ is the IC_50_ of the mutant peptide, IC50_WT_ is the IC50 of the wildtype (unmutated) peptide, R is the universal gas constant, and T is the temperature at 298K with R*T = 0.593 kcal/mol. For HwTx-IV mutations with IC_50_ values reported in both sources, the IC_50_ values reported by Minassian et al. [10] were used for performance analysis. The specific wildtype IC_50_ or pK_i_ measured in each study was used when converting that study’s mutational data into free energies.

### 4.3. Construction of NSAA Library of Tryptophan Analogues

NSAA analogues of tryptophan were created as follows. First, the “R-groups to Displace a water”, “Diverse R-groups”, “Aliphatic Monocyclic Rings” and “Aromatic Monocyclic Rings” libraries built-in to Maestro were enumerated at the 5, 6, and 7 positions on the indole ring of tryptophan. The enumeration was performed using the Custom R-Group Enumeration panel and the resulting NSAAs were imported into the Nonstandard Residues Panel, resulting in 582 tryptophan analogs. Additionally, a set of 48 commercially available tryptophan analogs were identified using SciFinder (Chemical Abstracts Service: Columbus, OH, USA) and also imported into the panel. In total, 630 NSAA analogues of tryptophan were created but only 617 were used for calculations to avoid duplicates.

### 4.4. WaterMap Calculations

The WaterMap simulations were performed as described previously [20] using the construct described above. As this WaterMap simulation was “holo,” ProTx-II was retained during the simulation.

### 4.5. MM-GB/SA Calculations and Analysis

MM-GB/SA calculations were set up using the Residue Scanning panel. The structure was imported into the Residue Scanning panel and “Stability and Affinity” calculation type was selected. For benchmarking MM/GB-SA performance with an implicit membrane, the Prime Membrane Setup panel was used to place an implicit solvent membrane on the structure. For ProTx-II, Chain F was chosen to bind to Chain B, and Chain G was chosen to bind to Chain F for HwTx-IV. Default settings of refinement were used for all Residue Scanning calculations along with a 0 Å cutoff for sidechain repacking. The job ran on 8 CPUs. Once Residue Scanning calculations for NSAA mutations were completed, the library was filtered for W24 mutations that had both ΔAffinity ≤ −5 and ΔStability < 0. Fifty-two resulting NSAA mutations were selected to be run in protein FEP at both Trp-5 and Trp-24. It has also been suggested previously that rescaling MM-GB/SA predicted affinities by a constant can allow for a better comparison to experimental data [25]. For the retrospective portion of the study, in addition to the unscaled MM-GB/SA affinities, re-scaled MM-GB/SA affinities were computed as well. To scale predicted binding affinities for MM-GB/SA, for each peptide predictions were divided by the slope of the regression line fit to a plot of ΔΔG_EXP_ vs. ΔΔG_MM-GB/SA_ [25].

### 4.6. FEP Calculations for GMT Mutations

All FEP mutation calculations were carried out as follows. For any NSAAs whose torsions were not already present in the OPLS3e forcefield, missing torsion parameters were fit prior to running FEP using the Force Field Builder panel in Maestro and merged into the default OPLS3e forcefield [35]. This customized forcefield was used for FEP calculations. FEP calculations were set up in the Protein FEP panel in Maestro. The equilibrated system file was imported and the Selectivity calculation type was selected. For ProTx-II, Chain F was chosen to bind to Chain B. For HwTx-IV, Chain G was chosen to bind to Chain F. Simulation parameters were 15ns simulation time and default lambda windows were used. The FEP job was run on 4 GPUs on a GPU cluster consisting of NVIDIA GeForce GTX 1080 and 1080ti GPU. For charged NSAAs such as homoarginine (hoR) and noraginine (norR), charge state was assigned directly within the Nonstandard Residues panel. For two mutations, ProTx-II[K26E] and ProTx-II[K26D], K26 was mutated into protonated Asp (ASH) and Glu (GLH), respectively. Using ASH and GLH has been shown to be important for accurate FEP calculations when the residue is in close proximity to another acidic side chain and there is a hydrogen bond network involved [25], which are both relevant in this case due to the close proximity to Glu-811, Asp-816, and Glu-818 on VSDII. Finally, for the separate set of FEP simulations which examined the effect of restraining the peptide and channel on iRMSD, position restraints were applied with a force constant of 1.0 (kcal/mol/ Å^2^) on all GMT and ion channel Cɑ atoms.

### 4.7. Calculation of iRMSD for GMT Pose Stability Analysis

To measure the stability of the GMT:VSD complex during the FEP trajectory, the average iRMSD during the last 5ns of the trajectory was calculated for each ProTx-II mutant using the Simulation Event Analysis panel. The RMSD was measured for the residues that make up the protein-protein interface. These residues included Trp-5, Met-6, Val-20, Arg-22, Leu-23, Trp-24, Lys-26, Lys-27, Lys-28, Leu-29, and Trp-30 on ProTx-II and Glu-760, Asn-764, Ala-767, Ile-768, Leu-771, Glu-811, Leu-812, Phe-813, Leu-814, Ala-815, Asp-816, Val-817, Glu-818, and Leu-820 on Na_V_1.7. These atoms were also used for superimposing the structures in each frame to the reference.

### 4.8. Statistics

Statistical analysis of the FEP results and their comparison to experimental data followed accepted best practices [36]. Notably, to minimize the effect of trial-to-trial variability in ΔΔG_FEP_, every mutation was run in triplicate with a different random seed and the ΔΔG_FEP_’s from each of the three independent simulations were averaged to arrive at a mean ΔΔG_FEP_ used in all analyses. Bootstrapped estimates for RMSE and MUE were calculated using the FEP+ panel software in Maestro. For binary classification, the AUC of a ROC plot and its associated CI was computed using Prism 8 with default options (GraphPad Software: San Diego, CA, USA). Mutations with a ΔΔG_EXP_ < 0 were classified as having a gain in potency.

## Figures and Tables

**Table 1:** FEP and MM-GB/SA performance overall and by category.

			FEP		MM-GB/SA	
Category	N	Min and Max ΔΔG in kcals/mol (Range)	RMSE	R^2^	RMSE	R^2^
Overall	47	−1.4–7 (8.4)	1.00 ± 0.1	0.66	3.89 ± 0.82	0.56
			**By Toxin**			
ProTx-II	10	−1.4–7 (8.4)	1.70 ± 0.16	0.78	7.57 ± 1.48	0.68
HwTx-IV	37	−1.09–1.88 (2.97)	0.69 ± 0.07	0.19	1.95 ± 0.64	0.24
			**By Property**			
Charge change mutations	24	−1.09–7 (8.09)	0.96 ± 0.15	0.81	3.75 ± 1.3	0.79
Neutral mutations	23	−1.4–1.88 (3.28)	1.02 ± 0.14	0.02	4.04 ± 1.3	0.23
			**By Position**			
Mutations facing into the membrane	19	−1.09–1.88 (2.97)	0.71 ± 0.10	0.31	1.65 ± 0.32	0.25
Mutations facing into the channel	16	−1.4–7 (8.4)	1.41 ± 0.15	0.70	6.4 ± 1.3	0.61
Mutations facing into the solvent	12	−0.96–0.68 (1.64)	0.53 ± 0.11	0.02	0.70 ± 0.21	0.03
